# Bioproducts From *Euglena gracilis*: Synthesis and Applications

**DOI:** 10.3389/fbioe.2019.00108

**Published:** 2019-05-15

**Authors:** Alexander Gissibl, Angela Sun, Andrew Care, Helena Nevalainen, Anwar Sunna

**Affiliations:** ^1^Department of Molecular Sciences, Macquarie University, Sydney, NSW, Australia; ^2^Australian Research Council Industrial Transformation Training Centre for Molecular Technology in the Food Industry, Sydney, NSW, Australia; ^3^Biomolecular Discovery and Design Research Centre, Macquarie University, Sydney, NSW, Australia

**Keywords:** *Euglena gracilis*, biosynthesis, dietary protein, vitamins, polyunsaturated fatty acids, wax esters, paramylon, large-scale cultivation

## Abstract

In recent years, the versatile phototrophic protist *Euglena gracilis* has emerged as an interesting candidate for application-driven research and commercialisation, as it is an excellent source of dietary protein, pro(vitamins), lipids, and the β-1,3-glucan paramylon only found in euglenoids. From these, paramylon is already marketed as an immunostimulatory agent in nutraceuticals. Bioproducts from *E. gracilis* can be produced under various cultivation conditions discussed in this review, and their yields are relatively high when compared with those achieved in microalgal systems. Future challenges include achieving the economy of large-scale cultivation. Recent insights into the complex metabolism of *E. gracilis* have highlighted unique metabolic pathways, which could provide new leads for product enhancement by genetic modification of the organism. Also, development of molecular tools for strain improvement are emerging rapidly, making *E. gracilis* a noteworthy challenger for microalgae such as *Chlorella* spp. and their products currently on the market.

## Background

The unicellular phototrophic protist *E. gracilis* is ubiquitous in most freshwater biotopes. It is capable of photoautotrophic (using sunlight), heterotrophic (using an external carbon source), and mixotropic (combining both modes) growth (Rodríguez-Zavala et al., 2010; Šantek et al., 2010; Buetow, 2011). Commercially relevant bioproducts synthesised by *E. gracilis* feature protein containing essential amino acids, pro(vitamins), lipids, and the β-1,3-glucan paramylon (Takeyama et al., 1997; Rodríguez-Zavala et al., 2010; Pollak et al., 2012).

*Euglena gracilis* has a natural ability to tolerate a number of external stresses, including acidic growth conditions and ionising radiation, and has been shown to be capable of heavy metal sequestration (Yamane et al., 2001; Hayashi et al., 2004; García-García et al., 2018). This physical endurance and metabolic adaptability may be harnessed for bioremediation of polluted water containing elevated levels of nitrogen, phosphates, organic carbon, Cd^2+^, Cr^3+^, Hg^2+^ Cr^6+^, Pb^2+^, uranium, and/or Zn^2+^ (Mahapatra et al., 2013; García-García et al., 2014).

*Euglena gracilis* can accumulate large amounts of the reserve polysaccharide paramylon, a β-1,3-glucan, which can constitute over 80% (w/w) of the dry weight (DW, biomass dried to a constant weight without oxidation) (Barsanti et al., 2001; Sun et al., 2018). Paramylon is uniquely produced by euglenoids, deposited as granules in the cytosol, and readily degraded and utilised as a carbon source under carbon starvation (Malkoff and Buetow, 1964; Kiss et al., 1986; Barsanti et al., 2001; Monfils et al., 2011). Paramylon and other β-1,3-glucans are of special interest because of their reported immunostimulatory and antimicrobial bioactivities (Kiss et al., 1986; Barsanti et al., 2001; Russo et al., 2017; Gissibl et al., 2018). Additionally, β-1,3-glucans have been shown to lower cholesterol levels and exhibit antidiabetic, antihypoglycemic and hepatoprotective activities; they have also been used for the treatment of colorectal and gastric cancers (Ooi and Liu, 2000; Kataoka et al., 2009; Barsanti et al., 2011).

Improvement of the performance of *E. gracilis* has mainly relied on developing cultivation conditions to favour the synthesis of the compound of interest, followed by scale-up of the cultivation volume, because of a general lack of genetic information on the metabolic pathways leading to the various bioproducts (O'Neill et al., 2015a; Wang et al., 2018). While a draft genome assembly and initial features of the genome have been made available, a complete annotated genome sequence is not on hand as yet (O'Neill et al., 2015a; Ebenezer et al., 2017). The large size and complexity of the *E. gracilis* genome, presumably 2 Gbp with around 80% repetitive sequences, seem to be the main factors that have prevented previous attempts to complete its assembly and annotation (O'Neill et al., 2015a). In the absence of complete genetic information, transcriptomic and proteomic studies have provided valuable insights into the complex metabolic pathways of *E. gracilis* and their regulation under different growth conditions (O'Neill et al., 2015b; Yoshida et al., 2016; Hasan et al., 2017).

There are a few isolated reports on genetic manipulation of *E. gracilis*. For example, transformation of *E. gracilis* chloroplasts with the gene coding for a cyanobacterial fructose-1,6-/sedoheptulose-1,7-bisphosphatase has been achieved by biolistic bombardment (Doetsch et al., 2001; Ogawa et al., 2015). Gene knockdown via RNA interference (RNAi) has led to the identification of the role of photoactivated adenylyl cyclase in phototaxis and the finding that glucan synthase-like 2 is essential for paramylon synthesis (Ntefidou et al., 2003; Tanaka et al., 2017). Recently, Khatiwada et al. reported a nuclear transformation platform facilitating further molecular genetic studies and the metabolic modification of *E. gracilis* (Khatiwada et al., 2019). The study showed that *Agrobacterium*-mediated transformation was superior to electroporation and biolistic bombardment, resulting in stable *E. gracilis* transformants. A number of *E. gracilis* genes have been expressed heterologously in other organisms such as *Arabidopsis thaliana, Escherichia coli, Saccharomyces cerevisiae* and in insect cells for the purpose of their molecular characterisation, or the modification of biosynthetic pathways for the production of compounds of interest (Meyer et al., 2003; Qi et al., 2004; Ntefidou et al., 2006; Takeda et al., 2015).

**Figure 1:**
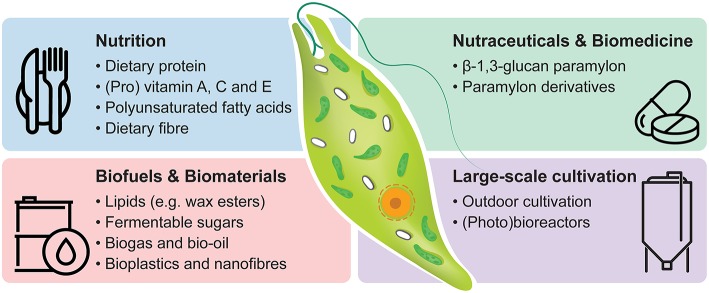
Industrial potential of *Euglena gracilis*. Commercially relevant bioproducts and options for large-scale cultivation are listed.

There is a growing interest in the commercial exploitation of *E. gracilis* based on the organism's versatility, resilience and capability to synthesise a broad and unique range of bioproducts (Suzuki, 2017). Here we present an overview of valuable bioproducts (e.g., dietary protein, provitamin A, vitamin C, vitamin E, lipids, and paramylon) and their cultivation condition-dependent synthesis in *E. gracilis* with special consideration to their industrial relevance and future development (Figure 1).

## Nutritional Value of *E. gracilis*

### Dietary Protein

There is a growing global demand for dietary protein with a high nutritional value due to the increasing human population in concurrence with socio-economic changes (Henchion et al., 2017). Microalgae can accumulate large quantities of proteins intracellularly and therefore represent an attractive alternative to more traditional sources of dietary protein like meat and fish (Henchion et al., 2017; Ritala et al., 2017). In this context, *E. gracilis* has been shown to produce protein containing all 20 proteinogenic amino acids (Isegawa et al., 1993; Rodríguez-Zavala et al., 2010; Hasan et al., 2017). Importantly, animal and *in vitro* studies have shown an excellent digestibility of protein derived from *E. gracilis* biomass (Hosotani and Kitaoka, 1977).

The mode of cultivation has a major impact on the total protein content of *E. gracilis* (Table 1), especially when comparing photoautotrophic (PT), heterotrophic (HT), and mixotrophic (MT) growth conditions. For example, HT growth in a medium supplemented with external nitrogen (ammonium) has resulted in the production of cell mass with a protein content of close to 0.7 g/g DW, whereas PT cultivation maximally yields 0.5 g/g DW (Chae et al., 2006; Rodríguez-Zavala et al., 2010). Regardless of the cultivation mode, dried *E. gracilis* cell mass would be competitive in terms of its protein content with fresh high-protein animal products such as beef, chicken or fish, of which the protein content usually does not exceed 0.4 g/g after cooking (Table 1). Therefore, protein sourced from *E. gracilis* could provide an alternative to supplement a vegetarian diet (US Department of Agriculture., 2018). The protein content of *E. gracilis* is on a par with popular microalgal food supplements such as those sourced from *Chlorella* spp. (Table 1) (de Oliveira et al., 1999; Tokuşoglu and Ünal, 2003; Ramazanov and Ramazanov, 2006;Rodríguez-Zavala et al., 2010).

**Table 1:** Comparison of the production titres of various bioproducts between *Euglena gracilis* and other source organisms or respective concentrations in alternative products.

**Bioproduct**	**Maximum production titre in *Euglena gracilis***	**Alternative source organism or product**	**Maximum production titre or concentration in alternative source organism or product**	**References**
Dietary protein	0.5 g/g DW; (continous PT cultivation); 0.7 g/g DW; (HT cultivation for 5 d)	*Chlorella* spp.; *Spirulina* spp. (grown for 15 d); Beef (lean, cooked); Chicken (cooked); Fish (cooked tuna)	0.6 g/DW; 0.7 g/DW; 0.4 g/g; 0.3 g/g; 0.3 g/g;	(de Oliveira et al., 1999; Tokuşoglu and Ünal, 2003; Chae et al., 2006; Ramazanov and Ramazanov, 2006; Rodríguez-Zavala et al., 2010; US Department of Agriculture., 2018)
Provitamin A (β-carotene)	3.5 mg/g DW or 3.5 mg/L culture; (PT cultivation); 1.4 mg/g DW or 11.4 mg/L culture; (MT cultivation); 3.4 mg/g DW or 71 mg/L culture; (MT/PT fed-batch two-step cultivation for >3 d)	Carrots, dehydrated	0.3 mg/g DW	(Takeyama et al., 1997; US Department of Agriculture., 2018)
Vitamin C (ascorbate)	4 mg/g DW or 8 mg/L culture; (PT cultivation for 1 d or 2 d); Negligible; (HT cultivation); 86.5 mg/L culture; (MT/PT fed-batch two-step cultivation)	Orange juice	50 mg/100 mL	(Shigeoka et al., 1980; Takeyama et al., 1997; Hasan et al., 2017; US Department of Agriculture., 2018)
Vitamin E (α-tocopherol)	2.6 mg/g DW or 8.6 mg/L culture; (PT cultivation for 3 d); mg/g DW or 44.2 mg/L culture; (HT fed-batch cultivation with ethanol for 18.5 d); 3.7 mg/g DW or 40 mg/L culture; (HT batch cultivation with ethanol for 5 d, bleached strain)	Wheat germ oil; Sunflower oil; Olive oil	1.5 mg/g; 0.4 mg/g; 0.1 mg/g	(Ogbonna et al., 1998; Rodríguez-Zavala et al., 2010; Grimm et al., 2015; Hasan et al., 2017; US Department of Agriculture., 2018)
PUFAs (DHA and EPA)	EPA: 5.8 mg/g DW; (MT cultivation for 4 d); DHA: 3.4 mg/g DW; (MT cultivation for 4 d)	Fish oil (e.g. from salmon)	0.2 g/g	(Barsanti et al., 2000; US Department of Agriculture., 2018)
Total lipids and WEs	0.2 g total lipids/g DW; (HT cultivation for 4 d and hypoxic cultivation for 6 d, mutant strain); 0.7 g total lipids/g DW; and 0.6 g WEs/g DW; (Anaerobic HT cultivation for 6 d using an elongase inhibitor)	*Botryococcus braunii*	0.8 g total lipids/g DW; (mostly FAs or similar compounds)	(Maxwell et al., 1968; Tucci et al., 2010; Yamada et al., 2016; Matos, 2017; Khan et al., 2018)
Paramylon	16 g/L culture; (HT repeated-batch cultivation for 17 d)	*Agrobacterium* sp. (grown for 3 d)	30 g/L culture of curdlan; (β-1,3-glucan)	(Yu et al., 2011; Šantek et al., 2012; Grimm et al., 2015)
Biogas (produced by anaerobic digestion)	650 mL/g DW; (after PT cultivation for 10 d); 800 mL/g DW; (after MT cultivation for 4 d)	*Chaetomorpha litorea*; *Chlamydomonas reinhardtii*; *Durvillaea antarctica*; *Macrocystis pyrifera*; *Scenedesmus obliquus*	80 mL/g DW; 590 mL/g DW; 180 mL/g DW; 180 mL/g DW; 290 mL/g DW	(Behera et al., 2014; Grimm et al., 2015)
Biomass	2–3 g DW/L culture; (PT cultivation for 3 d, shake flask); 12–13 g DW/L culture; (HT cultivation for 2 d, shake-flask); 23 g DW/L culture; (HT repeated-batch cultivation for 17 d, 5 L bioreactor); 48 g DW/L culture; (HT fed-batch cultivation for 7.6 d, 2 L bioreactor)	*Nannochloropsis* sp.; *Chlorella* sp.; *Crypthecodinium cohnii*	≥12 g/L culture; (PT cultivation, photobioreactor); 166 g/L culture; (HT fed-batch cultivation for 2.5 d, 6 L bioreactor); 109 g/L culture; (HT fed-batch cultivation for 16.7 d, 2 L bioreactor)	(Ogbonna et al., 1998, 2002; Richmond et al., 2003; Bumbak et al., 2011; Šantek et al., 2012; Grimm et al., 2015; Hasan et al., 2017)

*DHA, docosahexaenoic acid; DW, dry weight; EPA, eicosapentaenoic acid; FA, fatty acid; HT, heterotrophic; MT, mixotrophic; PT, photoautotrophic; PUFA, polyunsaturated FA; WE, wax ester*.

Initial transcriptomic studies indicated that amino acid synthesis pathways in *E. gracilis* were similar to those used by plants and bacteria (O'Neill et al., 2015b). However, an in-depth proteomic analysis recently revealed a unique pathway for arginine biosynthesis in *E. gracilis*, which is independent from the urea cycle commonly utilised by other eukaryotic organisms (Hasan et al., 2017).

### (Pro)Vitamin A, C, and E

Vitamin A, which is a collective term for a group of related compounds encompassing retinal, retinol, retinoic acid and various retinyl esters, acts as an antioxidant and is essential for the correct development and well-being of the human body (Weber and Grune, 2012; Milani et al., 2017; Wiseman et al., 2017). Deficiencies of this micronutrient can have severe health effects such as blindness in infants or an increased mortality rate during infections (Weber and Grune, 2012; Wiseman et al., 2017). Vitamin A is found only in animal products (e.g., fish and dairy) and its global human intake is currently supplemented to a large degree with plant-derived provitamin A (β-carotene). However, the metabolic conversion efficiency of β-carotene to vitamin A, generally considered to be 12:1, is dependent on several factors (e.g., bioavailability) and always needs to be taken into account in a dietary context (Weber and Grune, 2012).

Like other photosynthetic microorganisms, *E. gracilis* produces β-carotene as a protective pigment to ward off photo-oxidative damage to chloroplasts. Consequently, β-carotene titres per g DW are highest under PT cultivation conditions (Table 1). However, DW titres of *E. gracilis* under PT conditions are significantly lower than those obtained with MT/HT growth (Table 1) (Takeyama et al., 1997; Hasan et al., 2017). To circumvent this issue, a fed-batch two-step cultivation method with a transition from MT to PT growth conditions has been developed and shown to increase the β-carotene titre per L culture more than 6-fold (Table 1) (Takeyama et al., 1997).

The β-carotene content of *E. gracilis* cell mass grown in this two-step cultivation process or under PT conditions (3.4 or 3.5 mg/g DW, respectively) is exceptional, even in comparison with vegetables known to be high in β-carotene (Table 1). For example, dried *E. gracilis* cell mass contains up to 10-fold more β-carotene than dehydrated carrots (Table 1) (Takeyama et al., 1997; Weber and Grune, 2012; US Department of Agriculture., 2018). The recommended daily intake of vitamin A varies between countries, but is generally around 800 μg for an adult, which is equivalent to approximately 9.6 mg of the provitamin β-carotene or around 3 g of dehydrated *E. gracilis* cell mass with maximised β-carotene content (Takeyama et al., 1997; Weber and Grune, 2012). Therefore, *E. gracilis* cell mass can be considered an excellent vitamin A supplement and could improve a vitamin A-poor diet.

**Figure 2:**
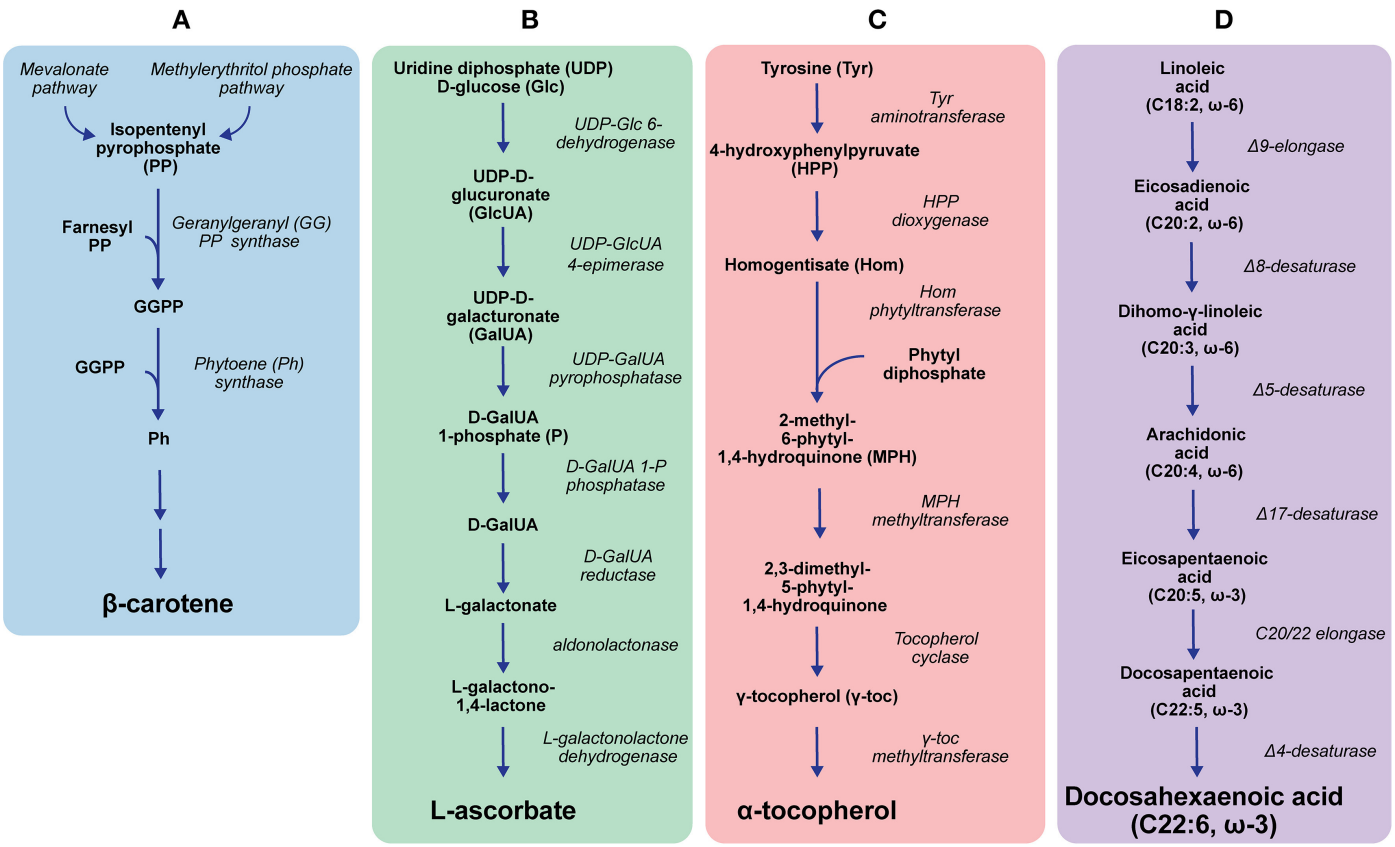
*Euglena gracilis* biosynthesis pathways of β-carotene **(A)**, ascorbate **(B)**, α-tocopherol **(C)**, and polyunsaturated fatty acids **(D)** (Shigeoka et al., 1992; Kim et al., 2004; Ishikawa et al., 2006; Ishikawa and Shigeoka, 2008; Lohr et al., 2012; Pollak et al., 2012; O'Neill et al., 2015b; Kato et al., 2016; Hasan et al., 2017). Only central enzymes/substrates/intermediates/products are shown.

A better understanding of the *E. gracilis* pathway for β-carotene biosynthesis (Figure 2A) could hold the key to further improve the yields. Two alternative metabolic routes to β-carotene have been identified biochemically or via transcriptomics in *E. gracilis*: the methylerythritol phosphate and the mevalonate pathway (Disch et al., 1998; Kim et al., 2004; O'Neill et al., 2015b). Many of the last enzymatic steps between the central intermediate isopentenyl pyrophosphate and the end product remain to be elucidated, as only the enzymes geranylgeranyl pyrophosphate synthase and phytoene synthase (see Figure 2A) have been verified by complementing the partially deleted carotenoid biosynthetic pathway of *Pantoea ananatis* expressed in *E. coli* with *E. gracilis* enzymes (Kato et al., 2016). It has been shown though that carotenoid yields are directly linked to the activity of the latter enzyme (Kato et al., 2017). However, more research is needed to determine if these enzymes could be a feasible target for strain improvement to increase β-carotene yields.

Vitamin C (ascorbate) is critical for human health as it acts as an antioxidant and co-factor for several enzymes involved in essential biosynthesis pathways (Grosso et al., 2013; Aghajanian et al., 2015). The unique ascorbate synthesis pathway from *E. gracilis* (Figure 2B) has been elucidated using radiotracer experiments, as well as transcriptomic and proteomic studies (Ishikawa and Shigeoka, 2008; O'Neill et al., 2015b; Hasan et al., 2017). At present, only two enzymes of this pathway, the D-galacturonate reductase and aldonolactonase, have been biochemically characterised *in vitro*. In an RNAi experiment, the latter enzyme has been shown to be essential for the ascorbate-dependent growth of the organism. Although these enzymes reportedly play a key role in *E. gracilis* ascorbate synthesis, a comparison between radiotracer studies indicated that the pathway flux is controlled by the epimerisation reaction catalysed by the uridine diphosphate D-glucuronate 4-epimerase (Shigeoka et al., 1979; Ishikawa et al., 2006; Ishikawa and Shigeoka, 2008). However, further studies are needed to confirm this proposed regulatory role.

PT cultivation induces the synthesis of ascorbate in *E. gracilis* as a mechanism to cope with reactive oxygen species (ROS) produced during photosynthesis (ROS scavenging). In addition, the enzyme ascorbate peroxidase reduces excess hydrogen peroxide to water by oxidation of ascorbate (Shigeoka et al., 1980; Ishikawa et al., 2010; Hasan et al., 2017). The typical ascorbate titre of a PT-cultivated *E. gracilis* is around 4 mg/g DW or 8 mg/L culture, whereas it is negligible for *E. gracilis* cultivated under HT conditions (Shigeoka et al., 1980; Hasan et al., 2017). The overall titre can be increased to 86.5 mg/L culture by the fed-batch two-step (MT to PT) cultivation method discussed above (Takeyama et al., 1997). It should be noted that ascorbate production by *E. gracilis* has not attracted much attention, probably because consumption of just 100 to 200 mL of juice from citrus fruits like oranges (Table 1) is enough to fulfil the daily recommended intake of adults (75 and 90 mg for females and males, respectively) (Grosso et al., 2013; US Department of Agriculture., 2018). Nevertheless, ascorbate and other bioproducts like vitamin E remain an important component of commercially available food supplements based on *E. gracilis* cell mass (Barsanti and Gualtieri, 2018).

Both α- and γ-tocopherol are, amongst β- and δ-, the major forms of dietary vitamin E. The α-form is one of the most abundant lipophilic antioxidants and considered more important for mammalian physiology. Adverse health effects, including neurological damage or anaemia, are associated with α-tocopherol deficiency (Niki and Traber, 2012). However, γ-tocopherol is the main dietary form in countries where consumed vegetable oils derive predominantly from soy and corn (e.g., in the USA), leading to an insufficient intake of α-tocopherol in these regions (Jiang et al., 2001; Rigotti, 2007; Traber, 2014).

*Euglena gracilis* has a plant-like α-tocopherol pathway, which has been confirmed by transcriptomic and proteomic studies. However, unlike higher plants, *E. gracilis* produces almost exclusively the α-form of tocopherol (Shigeoka et al., 1986; O'Neill et al., 2015b; Fritsche et al., 2017; Hasan et al., 2017). A key enzyme in its α-tocopherol synthesis pathway (Figure 2C) is γ-tocopherol methyltransferase (Shigeoka et al., 1986, 1992). This enzyme converts γ-tocopherol to α-tocopherol using *S*-adenosyl methionine (SAM) as the methyl group donor and also shows promiscuity towards β- and δ-tocopherol (Shigeoka et al., 1992). Compared to some γ-tocopherol methyltransferases from plants, the *E. gracilis* enzyme has a high K_M_ value (50 μM for SAM) and low specific activity (251 nmol/h per mg protein for γ-tocopherol) (Shigeoka et al., 1992; Koch et al., 2003). Yet, it is assumed that the conversion of γ-tocopherol to α-tocopherol is the rate-limiting step in the pathway of plants (Fritsche et al., 2017). Identifying what control mechanisms make *E. gracilis* so efficient in producing α-tocopherol could lead to strategies for the modification of higher plants to increase their α-tocopherol yields.

There are several factors influencing α-tocopherol levels in *E. gracilis*, for example, cultivation under light conditions or the addition of ethanol to the medium have been shown to increase α-tocopherol production (Table 1) (Kusmic et al., 1998; Fujita et al., 2008). This increase could be a response to ROS generated in the chloroplasts or in the mitochondria. Yet, the influence of light was shown to be independent of the presence of chlorophyll, suggesting that mitochondria may be primarily responsible for the regulation of α-tocopherol synthesis (Kusmic et al., 1998; Ogbonna et al., 1998; Fujita et al., 2008; Hasan et al., 2017). Long incubation times and supplementation of the medium with a carbon source (MT cultivation) have been shown to maximise the overall α-tocopherol yield due to an increase in the amount of cell mass. However, it has also been shown that α-tocopherol titres per g DW decrease under MT/HT cultivation (without ethanol) over time (Grimm et al., 2015; Hasan et al., 2017). These factors need to be taken into account when determining the most favourable time of harvest.

The technical set-up of a cultivation also plays an important role in α-tocopherol production, for example, hydrodynamic stress caused by fast stirring with baffled plates in a bioreactor cultivation has been shown to have a detrimental effect on the final yields of α-tocopherol during HT cultivation (Ogbonna et al., 1998). So far, the highest reported total α-tocopherol titres of 44.2 mg/L culture or 1.1 mg/g DW were achieved with *E. gracilis* in HT fed-batch cultivation after 455 h in a medium containing ethanol as a carbon source (Ogbonna et al., 1998). Comparable levels of 40 mg/L culture or 3.7 mg/g DW were reached after a relatively short incubation time of 120 h using a bleached strain in HT batch cultivation with a medium supplemented with ethanol, glutamate and malate (Rodríguez-Zavala et al., 2010). In contrast, some vegetable oils (e.g., olive, sunflower, and wheat germ oil) are marketed as good sources of daily vitamin E intake, but only have maximum α-tocopherol contents of 1.5 mg/g (Table 1), which further emphasises the commercial potential of *E. gracilis* as a viable candidate for industrial α-tocopherol production (Psomiadou et al., 2000; US Department of Agriculture., 2018). To the best of our knowledge, there are currently no reports of a commercial α-tocopherol production using *E. gracilis*.

### Polyunsaturated Fatty Acids (PUFAs)

PUFAs of the ω-3 or ω-6 series are defined by their first double bond at the position between the third and fourth or sixth and seventh carbon counted from the methyl end, respectively, and are considered essential for mammalian nutrition. In the Western world, major health conditions like cardiovascular and neurological diseases have been linked directly to a lack of long-chain ω-3 PUFAs, especially eicosapentaenoic acid (EPA) and docosahexaenoic acid (DHA), in the dietary intake (Deckelbaum and Torrejon, 2012; Innis, 2014). *Euglena gracilis* has a pathway for the synthesis of EPA and subsequently DHA (Figure 2D), and the enzymes within this pathway have been characterised biochemically. The proposed activity of Δ17-desaturase and C20/22 elongase (see Figure 2D) has been verified in *E. gracilis* cell extract only, whereas genes encoding the other enzymes (see Figure 2D) have been expressed heterologously (Wallis and Browse, 1999; Meyer et al., 2003; Qi et al., 2004; Damude et al., 2010; Pollak et al., 2012; Zhu et al., 2014).

Reported EPA and DHA titres in *E. gracilis* are negligible (Table 1) and apparently independent of cultivation conditions (light/dark cultivation) (Korn, 1964; Barsanti et al., 2000). These low titres could represent a good target for improvement by genetic engineering or non-recombinant metabolic engineering techniques based on the selection of improved phenotypes after the application of a selective pressure such as adaptive laboratory evolution (Portnoy et al., 2011; Dragosits and Mattanovich, 2013; Khatiwada et al., 2019). In an alternative approach, genes encoding single enzymes of the *E. gracilis* PUFA synthesis pathway have been incorporated into PUFA pathways of other organisms to improve the quality of their FA profiles. For example, the Δ8-desaturase from the *E. gracilis* pathway (see Figure 2D) has been incorporated into *A. thaliana* along with enzymes from other organisms for the production of EPA. In another report, the Δ4-desaturase from *E. gracilis* (see Figure 2D) was expressed together with the Δ15-desaturase from *Caenorhabditis elegans* in mammalian cells, resulting in a shift of the intracellular FA profile towards more valuable EPA and DHA (Qi et al., 2004; Zhu et al., 2014).

## Biofuels and Bioactive Compounds

### Wax Esters (WEs) and Other Lipids

Microalgae have been proposed as a source of feedstock for the production of biofuels such as biodiesel because of the high amounts of lipids, mostly FAs and WE, accumulated during the growth of some microalgal species (Matos, 2017). WEs can be used directly as biodiesel, whereas FAs must be processed first by esterification with an alcohol to yield WEs. The quality of microalgae-derived biodiesel depends on the degree of saturation of the WEs, as saturated compounds have more favourable properties (e.g., higher cetane number) (Ramos et al., 2009; Andreani and Rocha, 2012). Furthermore, there is a preference for the use of short- or medium-chain esters over long-chain esters in the industrial production of diesel and kerosene because shorter esters provide better cold flow properties and oxidative stability (Maurice et al., 2001). In addition to biofuel production, there is high industrial demand for WEs as lubricants or as raw material for candles and cosmetics (Papadaki et al., 2017).

In *E. gracilis*, WE synthesis serves as an electron sink for ATP production through glycolysis during anaerobiosis. Accordingly, the cells synthesise FAs, fatty alcohols (FAlcs) and ultimately WEs in larger quantities from paramylon under oxygen-limiting conditions (Inui et al., 1984; Tucci et al., 2010; Furuhashi et al., 2015; Yoshida et al., 2016). The central enzymatic steps of the pathway for WE formation in *E. gracilis* are catalysed by fatty acyl-CoA reductase (EgFAR) and wax synthase (EgWS). Wax ester synthase/diacylglycerol acyltransferase (EgWSD) isozymes have been shown to fulfil a role similar to that of EgWS. For example, RNAi-mediated silencing of EgWSD isoenzymes resulted in reduced wax ester production in *E. gracilis* (Teerawanichpan and Qiu, 2010; Tomiyama et al., 2017).

Heterologous expression of EgFAR together with EgWS in the industrially relevant organism *S. cerevisiae* has been shown to produce WEs of medium chain lengths from supplemented FAs. These findings highlight the potential of *E. gracilis* enzymes for the microbial production of biofuels (Teerawanichpan and Qiu, 2010; Tomiyama et al., 2017; Nandy and Srivastava, 2018).

The majority of the *E. gracilis*-derived lipids (WEs and FAs) are suitable for biodiesel production because they are saturated or have a low degree of unsaturation (Rosenberg, 1963; Tucci et al., 2010; Furuhashi et al., 2015). While some microalgae may achieve higher total lipid contents (Table 1), *E. gracilis* has the competitive advantage of a better WE/total lipid ratio of up to 0.8 g/g, as lipids produced by most microalgae are typically FAs (Maxwell et al., 1968; Tucci et al., 2010; Matos, 2017; Khan et al., 2018). Moreover, *E. gracilis* WEs are more suited for catalytic cracking for the production of fuel as the reaction is faster with less formation of unwanted poorly combustible polyaromatic compounds (Shimada et al., 2018). Enzymes of the FA biosynthesis pathway in *E. gracilis* have been shown to be crucial for the quality of WE derived from *E. gracilis*. For example, silencing of the genes coding for the *E. gracilis* 3-ketoacyl-CoA thiolase (EgKAT) isozymes EgKAT 1, EgKAT 2 and EgKAT 3 via RNAi lead to an increased production of short-chain WEs, and an artificial biodiesel inferred from the lipid composition before and after gene silencing indicated an improvement of cold flow properties (Nakazawa et al., 2015).

The lipid yield and the ratio of FAs/FAlcs to WEs have been shown to be strongly dependent on the cultivation conditions and the *E. gracilis* strain used (Tucci et al., 2010). As yet, a maximum total lipid and WE titre of ~0.7 and 0.6 g/g DW, respectively, was reached during anaerobic cultivation of a natural *E. gracilis* isolate with the addition of an elongase inhibitor (Tucci et al., 2010).

Furthermore, substantial research has been performed to improve the WE yields specifically. For example, a multi-step cultivation process has been developed on a laboratory scale (in flasks) aiming to maximise WE synthesis. In the first step of this process, *E. gracilis* is cultivated under inexpensive PT conditions (Arashida and Mitra, 2015). Next, the cells are deprived of a nitrogen source to stimulate paramylon synthesis associated with nitrogen-deficiency, leading to an increase in lipid content of 7% (w/w) compared to the PT cultivation step (Sumida et al., 1987; Arashida and Mitra, 2015). In the third step, WE synthesis from paramylon is induced by changing to anaerobic cultivation. This is followed by the extraction of the WEs with an organic solvent and column purification, resulting in the production of a source of biofuel (Arashida and Mitra, 2015; Matos, 2017).

In recent years, a growing number of cytometry approaches have been developed for the screening of *E. gracilis* mutant strains with an increased lipid content. For example, intracellular lipids of a mutant strain generated by Fe-ion irradiation of the WT parental strain were stained with a boron-dipyrromethene dye (BODIPY^505/515^) to isolate high-producers using fluorescence-activated cell sorting. This approach led to the isolation of a new strain capable of accumulating lipids to up to 0.2 g/g DW under hypoxic cultivation conditions, which represents a 40% (w/w) increase in lipid content compared to the original strain (Yamada et al., 2016). Other current cytometry-based techniques are capable of identifying phenotypes of single cells associated with a high lipid content directly. Two examples are high-throughput optofluidic image cytometry, where lipid-overproducing mutants can be identified without the need for staining of the cells with potentially phenotype-altering dyes, and fluorescence imaging flow cytometry, which allows the normalisation of fluorescence values against the cell size to avoid false-negative results (i.e., mislabelling of small cells with relatively high fluorescence values) (Lei et al., 2016; Muñoz et al., 2018).

Besides random mutagenesis, targeted silencing of single genes has shown potential for improving WE yields. For example, knock-down of the starch degradation kinases 1 and 2 using RNAi was shown to lead to a 1.2 and 1.4-fold increase in WE accumulation, respectively, under anaerobic growth conditions (Kimura and Ishikawa, 2018). As another target for a metabolic engineering approach, *trans*-2-enoyl-CoA reductase has been identified as a potentially rate-limiting enzyme in the FA synthesis pathway of *E. gracilis* (Inui et al., 2017).

### Paramylon

#### Biosynthesis of Paramylon

**Figure 3:**
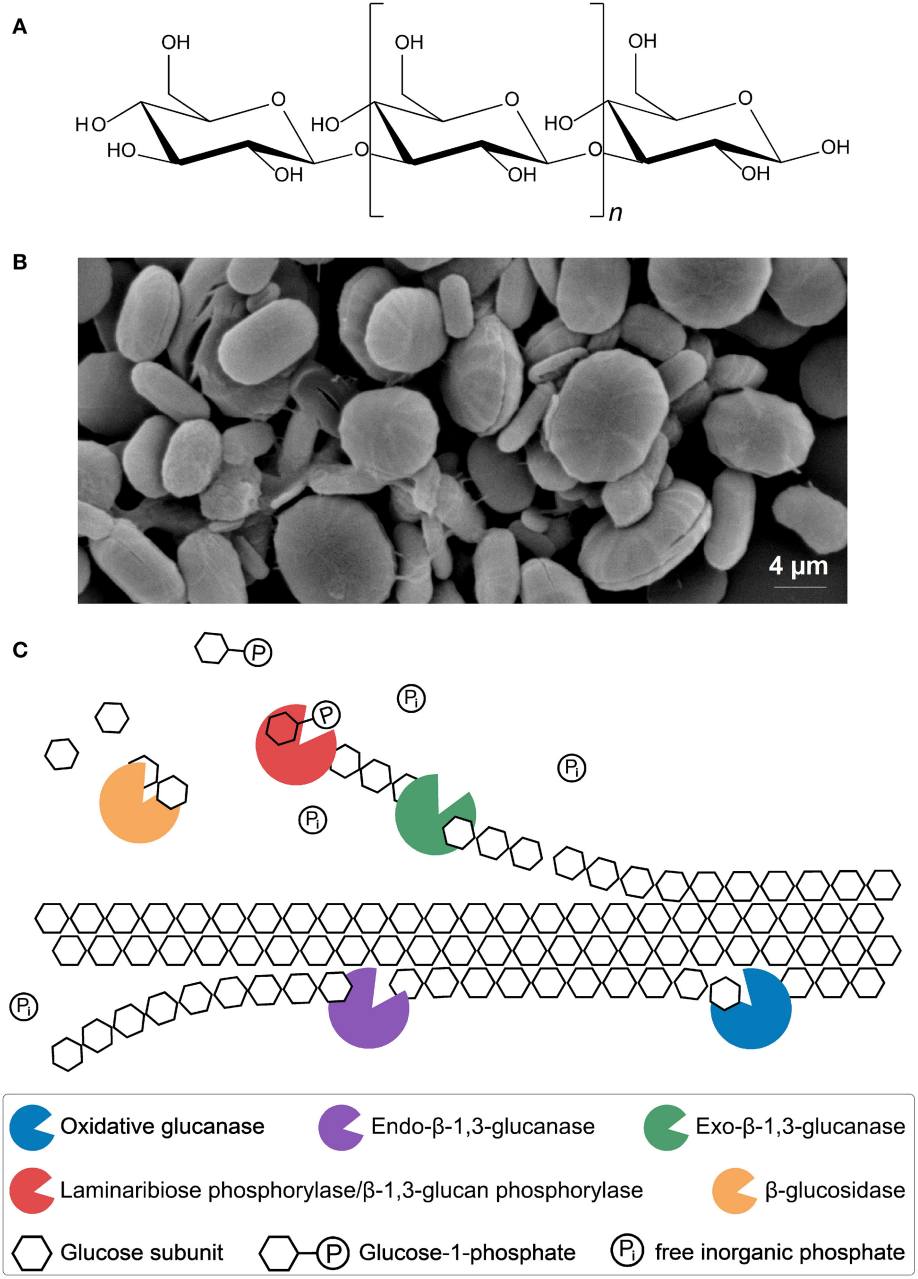
Molecular structure of paramylon **(A)**. β-1,3-glucan chain (~700 ≤ *n* ≥ 3,000) (Miyatake and Kitaoka, 1983; Koizumi et al., 1993; Barsanti et al., 2011). Microscopic image of paramylon granules **(B)**. Shown are ellipses and rods without biomembrane (Bäumer et al., 2001; Monfils et al., 2011). Putative enzymatic mechanism for synergistic paramylon degradation in *Euglena gracilis* **(C)**. Enzymes, substrates and products are shown. Oxidative glucanases: cleavage of crystalline paramylon to make it accessible for other enzymes, possibly similar to oxidative cleavage of cellulose (Johansen, 2016). Hydrolytic endo-β-1,3-glucanases: random cleavage of the polysaccharide chain (Takeda et al., 2015). Hydrolytic exo-β-1,3-glucanases and β-glucosidases: oligoglucans and single glucose units are cleaved off, respectively, at the ends of accessible and freed polysaccharide chains, comparable to the cellulolytic enzymes from *Trichoderma reesei* (Barras and Stone, 1969; Jeng et al., 2011; Keshavarz and Khalesi, 2016). Laminaribiose phosphorylase/β-1,3-glucan phosphorylase: cleaving of laminaribiose/laminarioligosaccharides into glucose/laminarioliosaccharides and glucose-1-phosphate, which requires free inorganic phosphate (Marechal, 1967; Kuhaudomlarp et al., 2018).

Paramylon is the water-insoluble storage polysaccharide of *E. gracilis*, which consists of β-1,3-linked glucose subunits, and has an estimated molecular weight between 100 and 500 kDa (Figure 3A) (Miyatake and Kitaoka, 1983; Koizumi et al., 1993; Barsanti et al., 2011). Paramylon molecules are arranged as an intermolecular triple helix forming microfibrils, which in turn make up fibres. Rectangular and wedge-shaped segments consisting of these fibres are arranged to form granules, which can be synthesised by *E. gracilis* in different shapes like ellipses or rods (Figure 3B). The granules are ~1–6 μm long, surrounded by a biomembrane and show an unusual high degree of crystallinity, setting them apart from other carbohydrate storage products found in plants and algae (Miyatake and Kitaoka, 1983; Koizumi et al., 1993; Bäumer et al., 2001; Barsanti et al., 2011; Monfils et al., 2011).

*E. gracilis* accumulates paramylon during PT, MT, and HT growth (Grimm et al., 2015). Cultivation under strict HT conditions leads to increased paramylon levels during the exponential growth phase, while light has been shown to be detrimental to the accumulation and conservation of paramylon under MT growth, probably because the metabolic switch to paramylon degradation is under the influence of a photoreceptor (Kiss et al., 1986; Barsanti et al., 2001). One of the highest paramylon titres reported (16 g/L culture) was obtained in a repeated-batch cultivation under HT conditions in the dark (Table 1), using a medium supplemented with potato liquor, vitamins, and a high concentration of glucose (30 g/L) (Šantek et al., 2012; Grimm et al., 2015).

#### Paramylon as Feedstock for Biomaterials and Biofuels

Various industrial applications have been proposed for paramylon, including its use as a substrate for thermoplasticisation. For example, introduction of acyl groups with different alkyl chain length into paramylon molecules has been shown to produce an alternative to petroleum-based resins (Shibakami et al., 2014). Another example is the production of self-assembling β-1,3-glucan nanofibres derived from paramylon (Shibakami et al., 2013a). The nanofibres can be surface-modified with functional groups (e.g., carboxylic acid) and could serve as stimuli-responsive polymers or drug delivery systems (Shibakami et al., 2013b). Also, it has been suggested to use paramylon myristate as an “all-natural” pressure-sensitive adhesive (Shibakami and Sohma, 2018).

Microalgal biomass is considered a third-generation biofuel feedstock, not only because of its potentially high lipid content, but also because of the high amounts of complex carbohydrates (e.g., cellulose) that can be produced by many algal species (Lee and Lavoie, 2013). These complex carbohydrates can be hydrolysed chemically into their constituent monosaccharide units (e.g., glucose) for subsequent fermentation to bioethanol, or alternatively using enzymes, as harsh chemical conditions can interfere with fermentation (Mussatto et al., 2010; Chen et al., 2013; Al Abdallah et al., 2016). In principle, enzymatic hydrolysis and conversion processes could be applied to paramylon. However, paramylon granules have been shown to be recalcitrant to enzymatic degradation and it is very likely that a consortium of different enzymes is required for the efficient degradation of this polysaccharide (Sutivisedsak et al., 2013). So far, only two *E. gracilis* enzymes involved in paramylon degradation have been characterised, an endo-β-1,3-glucanase and a laminaribiose phosphorylase (LBP)/β-1,3-glucan phosphorylase (β-1,3-GP) involved in paramylon degradation have been characterised (Vogel and Barber, 1968; Takeda et al., 2015; Kuhaudomlarp et al., 2018). A putative enzymatic mechanism for complete synergistic hydrolysis of the polysaccharide paramylon to glucose is shown in Figure 3C.

Other organisms, mostly belonging to the fungal genera *Trichoderma* and *Aspergillus*, have been explored as alternative sources for paramylon-degrading enzymes. For example, a fractionation of enzymes secreted by *T. harzianum* Rifai PAMB-86 led to the enrichment of β-1,3-glucanases able to degrade paramylon to some degree, yielding mostly glucose (Giese et al., 2011; Sutivisedsak et al., 2013).

Biogas and bio-oil can also be produced from microalgal carbohydrates via thermochemical conversion processes like gasification at high temperatures or pyrolysis in the absence of oxygen, respectively (Behera et al., 2014). Another way to produce biogas consisting mostly of methane from microalgal biomass is through anaerobic digestion by a consortium of microorganisms. *Euglena gracilis* cell mass has been shown to be a suitable source for the production of biogas by anaerobic digestion, producing around 650 or 800 mL biogas/g DW under PT or HT cultivation conditions, respectively (Grimm et al., 2015). These yields were up to 10-fold higher than those obtained from microalgae such as *Chaetomorpha litorea, Chlamydomonas reinhardtii, Durvillaea antarctica, Macrocystis pyrifera*, and *Scenedesmus obliquus* (Table 1) (Behera et al., 2014).

At this time, microalgae-derived biofuels are not economically feasible because they are not competitive with fossil fuels due to their higher production costs. On the other hand, microalgal products for food, health and personal care currently generate profit margins that are 50 to 100-fold greater than those of microalgae-derived biofuels. Consequently, it may be a viable commercial strategy to use *E. gracilis* to produce these high-value products with biofuel commodities as a co-product (Barsanti and Gualtieri, 2018).

#### Application of Paramylon in Nutrition and Biomedicine

There are several health benefits associated with β-glucans, including immunostimulatory and antioxidant effects (Barsanti et al., 2011). They also act as a dietary fibre and have been shown to lower blood cholesterol levels (Nakashima et al., 2018b). Mouse and rat studies, as well as experiments with different mammalian cell lines have been performed to determine the potential impact of the β-1,3-glucan paramylon on human health. For example, mice fed with 2% (w/w) paramylon in their diet and challenged with the (human) influenza virus A/PR/8/34 (H1N1) showed higher survival rates and cytokine levels (IFN-γ IL-1β, IL-6, IL-10, and IL-12) compared to the control group in concurrence with lower virus titres, suggesting that paramylon served as an effective regulator of the immune response providing protection against the virus (Nakashima et al., 2017). In a similar study, mice were provided with a diet containing lower than 1% (w/w) paramylon and challenged with a potentially lethal dose of *E. coli*. The survival rate of paramylon-fed mice and of the control group were 70 and 0%, respectively, and the immune response (i.e., antibody titres, IL-2 production, natural killer cell cytotoxicity, and phagocytosis activity) was significantly increased in the group fed with paramylon. The performance of paramylon in this study was as effective as or more effective than two commercially available β-glucan products for animal feed that were derived from yeast (Levine et al., 2013).

Furthermore, there are effects attributed to paramylon beyond the immune response to pathogens. For example, mice treated with 2,4,6-trinitrochlorobenzene, which would usually induce atopic dermatitis (AD)-like skin lesions, were fed with paramylon at 1% (w/w) of their diet. Paramylon inhibited the development of AD-like skin lesions, reduced cytokine (IFN-γ, IL-4, IL-12, and IL-18) levels and dermatitis scores. Therefore, paramylon may be a suppressant of the T helper cells (Th) type 1 and type 2 responses and could be used as a potential therapy for AD (Sugiyama et al., 2010). Another indicator that paramylon has regulatory effects on the cells was a study on a collagen-induced arthritis mouse model (for rheumatoid arthritis), where 2% (w/w) paramylon in the diet relieved arthritis symptoms and decreased cytokine (IFN-γ, IL-6, and IL-17) levels, so the authors concluded a possible involvement of Th type 17 cells (Suzuki et al., 2018). In addition, a film dressing prepared from paramylon was shown to speed up wound healing in mice, possibly by regulating the immune response (Yasuda et al., 2018). Paramylon also can act as a potent antioxidant protecting mice from acute hepatic injury induced by CCl_4_ treatment and was shown to alleviate non-alcoholic steatohepatitis in mice caused by a combination of streptozocin injection and high fat diet when orally administered at a dosage of 1 or 3 g/kg bodyweight (BW) per day, respectively (Sugiyama et al., 2009; Nakashima et al., 2018a). Moreover, feeding male rats with 20 mg paramylon/kg BW daily was shown to improve the quality of their sperm (e.g., motility, vitality, and acrosome integrity) (Ak Sonat et al., 2018).

Remarkably, paramylon has been shown to reduce the risk of cancer. For example, when preneoplastic aberrant crypt foci (marker for colon cancer risk) were induced in the colon of mice by 1,2-dimethylhydrazine treatment, the subsequent inclusion of 2% (w/w) paramylon into their diet reduced the development of colon cancer by 50% (Bird, 1995; Watanabe et al., 2013). The mechanisms responsible for this observed reduction are still unclear, but may be related to an effect of paramylon on the gut microbiome (Watanabe et al., 2013).

Until now, the research on the health benefits of paramylon has focussed mainly on applications to treat human conditions, but it has been suggested that it also could be beneficial to livestock and fish health. Studies undertaken with porcine leucocytes, chickens (broilers) and fishes (e.g., rainbow trout, Nile tilapia, and red drum) have shown that supplementation of the medium with paramylon or inclusion of paramylon in a diet resulted in immunostimulatory and/or -regulatory activity on the cells or animals, with positive effects such as host protection against parasites (Sonck et al., 2010; Skov et al., 2012; Levine et al., 2018; Yamamoto et al., 2018a,b). However, more studies would be needed to elucidate the nature of the observed effects and to confirm quantifiable benefits for the animals.

Chemical derivatives of paramylon (activated paramylon) have been shown to exhibit augmented or novel bioactivities. For example, the antimicrobial activity of paramylon was enhanced chemically by introducing positively charged groups (e.g., 2-hydroxy-3-trimethylammoniopropyl, N,N-diethylaminoethyl, and N,N-dimethylaminoethyl groups) and sulphated paramylon was shown to exhibit anti-HIV activity (Sakagami et al., 1989; Koizumi et al., 1993). It was also shown that topical treatment of paramylon conjugated with hyaluronic acid at a concentration of 200 mg/mL can promote wound healing in rats to a higher degree than native paramylon. As a result, corneal epithelial cell migration was increased and the acute inflammatory reaction caused by corneal alkali burns *in vivo* was supressed (Choi et al., 2013). Furthermore, it was shown *in vitro* and in a mouse model that straight-chain cationic 2-hydroxy-3-(trimethylammonio)propyl paramylon (HTAP) is capable of effectively sequestering bile salts, indicating an anti-diabetic effect, and the bodyweight of obese mice was reduced when the feed was supplemented with 2% (w/w) HTAP (Shibakami et al., 2018).

Incubation of originally granular paramylon with bases like sodium hydroxide has been shown to yield soluble nanofibers. There are strong indications that this pretreatment of paramylon has the effect of an increase in stimulation of leucocytes and hepatoprotection, probably because the β-1,3-glucan triple-helices of paramylon are disrupted in the process and the apparently more bioactive single helices are thereby exposed (Kataoka et al., 2002; Kusmic et al., 2018). Accordingly, alkalised paramylon was shown to upregulate proinflammatory factors (COX-2, IL-6, NO, TNF-α and translocation of NF-κB) in human lymphomonocytes at a higher rate than the similarly treated commercial β-glucan product MacroGuard, which is derived from *S. cerevisiae* (Russo et al., 2017).

To maximise the bioactive potential of paramylon, the granules could be hydrolysed to soluble shorter-chain β-1,3-glucans, which may lead to an increased blood plasma availability and a stronger or different immune response compared to an insoluble preparation (Rice et al., 2005). Only recently, it has been shown that microwave pretreatment of the granules can be used to enhance the activity of paramylon-degrading enzymes, yielding soluble immunostimulating hydrolysis products (Gissibl et al., 2018).

The *in vitro* synthesis of soluble β-1,3-glucans could be an alternative to the hydrolysis approach. However, the current chemical synthesis of oligosaccharides is still laborious and product yields are very low despite some advances in the field, whereas enzymatic synthesis has been considered as a technically feasible option (Plante et al., 2001; Ogawa et al., 2014). Towards this end, an *E. gracilis* β-1,3-glycosyl transferase (paramylon synthase) complex has been purified, characterised, and shown to convert uridine diphosphate D-glucose into β-1,3-glucan. Unfortunately, the product yield of this reaction has not been reported (Bäumer et al., 2001). Alternatively, the reversal of the equilibrium reaction of the LBP/β-1,3-GP (see Figure 3) has been proposed as a way of synthesising short-chain β-1,3-glucans, using initially glucose and subsequently glucose-1-phosphate as substrates (Kitaoka et al., 1993; Kuhaudomlarp et al., 2018). Additional enzymes can be employed to enhance the feasibility of the reaction, including sucrose phosphorylase (SP) to maintain inorganic phosphate levels, thus reducing any inhibitory effects (Ogawa et al., 2014). In a further development of this system, the reusability of LBP/β-1,3-GP was demonstrated following covalent immobilisation onto solid support enzyme carriers or formation of cross-linking of enzyme aggregates. However, the product (laminaribiose) yield of the immobilisation approach in combination with the SP enzyme was just 20% (w/w) (Müller et al., 2016).

Despite substantial evidence for the health-enhancing bioactivities of paramylon and paramylon-derived compounds as outlined above, we are not aware of any clinical studies confirming these claims. By definition, paramylon and its related compounds can only be considered 'nutraceuticals' and not 'pharmaceuticals', which has restricted them to be used only as food supplements (Santini et al., 2018). Currently, the health-enhancing properties of paramylon are exploited in this way by several new or already established companies in Japan and the United States, which are selling paramylon-rich *E. gracilis* whole cell meal or extracted paramylon (Barsanti and Gualtieri, 2018).

## Large-Scale Cultivation of *E. gracilis*

### Towards Industrial-Scale Cultivation

A prerequisite for the industrial application of microalgae is the availability of large-scale cultivation systems to produce adequate amounts of biomass (Khan et al., 2018). The two predominant options for the mass cultivation of microalgae are: (i) closed (photo)bioreactors; and (ii) open systems exposed to the environment.

Preliminary laboratory-scale experiments to determine *E. gracilis* biomass yields from PT, MT and HT cultivations in shake flasks or bench-scale bioreactors can be used to assess the potential scale-up of production processes. HT cultivation with glucose as a carbon source in shake flasks usually reaches DW titres of ~12 g/L culture, whereas repeated-batch cultivation supplemented with potato liquor and glucose in a bioreactor (5 L working volume) was shown to reach ~23 g/L culture (Šantek et al., 2012; Grimm et al., 2015; Hasan et al., 2017). A maximum *E. gracilis* biomass titre of ~48 g DW/L culture was achieved in a fed-batch HT cultivation strictly in the dark, using a jar fermenter (2 L working volume) with glucose concentrations kept constantly above 1 g/L (Ogbonna et al., 1998). As some microalgal species, including *Chlorella* sp. and *Crypthecodinium cohnii*, have been shown to reach more than twice the DW of *E. gracilis* under HT cultivation (Table 1), the current *E. gracilis* HT cultivation process should be revised to improve its efficiency (Bumbak et al., 2011).

DW titres per L of MT cultures have been shown to fall short of HT cultures in comparative experiments by around 30% (w/w), although it has been suggested that the PT and HT metabolisms could complement each other under MT conditions (Ogbonna et al., 2002; Hasan et al., 2017). PT cultivation produces ~20-fold less biomass (between 2 and 3 g DW/L culture) compared to HT and MT cultivation (Table 1) (Grimm et al., 2015; Hasan et al., 2017). Attempts to enhance *E. gracilis* biomass production under PT conditions, for example with the use of a photobioreactor, resulted in maximum titres similar to those of shake flask cultures, which are several times lower than those of some microalgal species grown under closed PT conditions (Table 1) (Richmond et al., 2003; Chae et al., 2006; Hasan et al., 2017).

Outdoor PT cultivation is considered the most economical option for the bulk production of microalgal biomass (Apel and Weuster-Botz, 2015). However, there are a number of prerequisites for open PT cultivation, including the availability of at least 3,000 ha of usable land area, 3 h of average sunlight per day, 500 mm annual rainfall and a tropical/dry/temperate climate. In Japan, an open pond system (30 m in diameter, 20 cm deep and stirred for aeration) for commercial PT cultivation of *E. gracilis* has been shown to be economically feasible and it can be expected that outdoor systems would be easily implemented in other countries such as Australia, Brazil, Malaysia, Thailand, the USA and Vietnam (Suzuki, 2017). Open ponds are low-tech solutions and potentially cheaper when compared to technically more demanding open systems like raceway tanks, which in turn promise higher yields (Apel and Weuster-Botz, 2015). In a laboratory set-up mimicking local environmental conditions, experiments with a raceway for the PT cultivation of *E. gracilis* resulted in biomass yields close to calculated theoretical values. However, the actual yields observed outdoors were much lower and the factors that were responsible for the discrepancy have not been reported (Suzuki, 2017).

### Challenges and Possible Solutions

Reports on industrial-scale HT cultivation of *E. gracilis* in controlled bioreactors have been only conceptual so far. For example, Levine et al. have proposed a multi-stage process where smaller bioreactors generate the inocula for the bigger stages (up to 1,000,000 L) with the aim to increase biomass titres to 120 g/L culture or higher (Levine et al., 2013). This biomass yield would be comparable to or higher than those obtained with some microalgae grown under HT conditions (Table 1) (Bumbak et al., 2011). However, there is no further information on whether this proposed system was ever built and tested. It is likely that certain disadvantages of HT cultivation prevented its realisation such as the cost of the medium containing an organic carbon source like glucose (Šantek et al., 2010). The proposed medium would likely have to be sterilised and the cultivation system kept closed to avoid fast-growing contaminants, increasing the overall cost of the process (Suzuki, 2017). The use of wastewater, municipal organic waste, compost or nutrition-rich waste products from the food industry such as potato liquor or dairy effluent could lower the costs by replacing or supplementing the HT growth medium with the additional benefit of potentially bioremediating these effluents (Šantek et al., 2012; Mahapatra et al., 2013; Yadavalli et al., 2014; Torihara and Kishimoto, 2015; Tossavainen et al., 2018).

PT cultivation on the other hand, although being a feasible alternative to HT cultivation, is restricted by low yields even after the technical optimisation of the cultivation process (Chae et al., 2006; Grimm et al., 2015; Hasan et al., 2017; Suzuki, 2017). A solution to this problem could be the genetic modification (GM) of *E. gracilis* to improve biomass and/or bioproduct yields. For example, expression of the *Synechococcus elongatus* PCC 7942 fructose-1,6-/sedoheptulose-1,7-bisphosphatase gene in *E. gracilis* chloroplasts was shown to significantly enhance its photosynthetic activity, as well as the paramylon yield (by almost 2-fold), the WE yield under anaerobic conditions (~100-fold) and the biomass yield (~2-fold) (Ogawa et al., 2015). However, local legal regulations could prevent (outdoor) cultivation of genetically modified *E. gracilis* strains (Beacham et al., 2017). Alternatively, conventional mutagenesis could be used to generate 'natural' mutant strains with desired traits, a process which is generally not considered GM. Several reports have described the successful mutagenesis (e.g., by irradiation) of *E. gracilis* (Schiff et al., 1980; Yamada et al., 2016; Suzuki, 2017).

A major limitation facing the industrial-scale production of *E. gracilis* biomass is vitamin auxotrophy. Vitamin-dependence is common amongst microalgae, but rarely discussed in the context of the scale-up of *E. gracilis* cultivation (Croft et al., 2006). *Euglena gracilis* shows an absolute requirement for vitamins B_1_ (thiamine) and B_12_ (cobalamin), so complex *E. gracilis* media usually contain at least 50 ng/L and 10 ng/L, respectively, of these compounds (Isegawa et al., 1987; Shigeoka et al., 1987; Croft et al., 2005; Hasan et al., 2017). Accordingly, the supplementation of both vitamins, produced either by chemical synthesis (thiamine) or extracted from bacterial sources (cobalamin), would be a factor in a scaled-up *E. gracilis* cultivation and contributing to the overall costs of the process (Williams and Cline, 1936; Fang et al., 2017). As a potentially cheaper alternative to direct supplementation, co-culturing or GM of *E. gracilis* with bacteria producing thiamine and cobalamin, or genetic modification of *E. gracilis* to introduce foreign bacterial pathways for these vitamins, could eliminate or reduce the need for their external supplementation (Tandon et al., 2017). Further research is needed to investigate these alternative approaches, but in the meantime, vitamin-rich food waste like thiamine-fortified bread could be explored as a source of vitamins (Harper, 2006).

## Conclusions

There is an emerging market for *E. gracilis*-derived products, which is reflected in the recent founding of new companies specialised in the cultivation and commercialisation of *E. gracilis*. PT cultivation of *E. gracilis* is currently favoured, probably due to lower cost and relative ease of set-up, but HT cultivation for higher yields of cell mass and paramylon could soon become reality. Presently, only nutritious whole cell meal, the nutraceutical paramylon and cosmetics are commercially viable, while biofuel production from *E. gracilis* cell mass is in contrast not yet feasible. However, carbohydrate and lipid yields in *E. gracilis* can be improved by new cultivation technologies and metabolic engineering approaches, possibly providing an affordable alternative to fossil fuels in the near future.

## Author Contributions

Original data collection and draft preparation by AG. All authors listed have made a direct and intellectual contribution to the work and approved it for publication.

### Conflict of Interest Statement

The authors declare that the research was conducted in the absence of any commercial or financial relationships that could be construed as a potential conflict of interest.
